# The Association between Coffee Consumption and Risk of Colorectal Cancer in a Korean Population

**DOI:** 10.3390/nu13082753

**Published:** 2021-08-11

**Authors:** Youngyo Kim, Jeonghee Lee, Jae Hwan Oh, Hee Jin Chang, Dae Kyung Sohn, Aesun Shin, Jeongseon Kim

**Affiliations:** 1 Department of Food and Nutrition/Institute of Agriculture and Life Science, Gyeongsang National University, Jinju 52828, Korea; youngyokim@gnu.ac.kr; 2 Department of Cancer Biomedical Science, Graduate School of Cancer Science and Policy, National Cancer Center, Goyang 10408, Korea; jeonghee@ncc.re.kr; 3 Center for Colorectal Cancer, National Cancer Center Hospital, National Cancer Center, Goyang 10408, Korea; jayoh@ncc.re.kr (J.H.O.); heejincmd@ncc.re.kr (H.J.C.); gsgsbal@ncc.re.kr (D.K.S.); 4 Department of Preventive Medicine, Seoul National University College of Medicine, Seoul 03080, Korea; shinaesun@snu.ac.kr

**Keywords:** coffee, colon cancer, rectal cancer, case-control study, coffee additives

## Abstract

This study was performed to investigate the association between coffee consumption and risk of colorectal cancer in a Korean population and examine whether the association can be altered by adjustment for intake of coffee additives. We conducted a case-control study involving 923 colorectal cancer cases and 1846 controls matched by sex and age (within 5 years). A semi-quantitative food frequency questionnaire was used to assess coffee intakes. High coffee consumption was associated with lower odds of developing colorectal cancer (≥3 cups/day vs. no drinks, OR = 0.68; 95% CI: 0.49–0.96). When we additionally controlled for consumption of coffee additives including sugar and cream, the inverse association became stronger (≥3 cups/day vs. no drinks, OR = 0.22; 95% CI: 0.14–0.33), and a significant inverse linear trend was shown (*P*_trend_ < 0.0001). The inverse associations were observed for proximal (*P*_trend_ = 0.0001) and distal (*P*_trend_ = 0.0003) colon cancer, and rectal cancer (*P*_trend_ < 0.0001) in the stratified analysis by anatomical sub-sites. Regarding sex, inverse associations between coffee consumption and colorectal cancer were found for men (*P*_trend_ < 0.0001) and women (*P*_trend_ = 0.0021). In the stratified analysis by obese status of subjects, inverse linear trends were observed in both non-obese and obese people (*P*_trend_ < 0.0001). High coffee consumption may be associated with a lower risk of colorectal cancer in the Korean population and the degree of decrease in the odds of developing colorectal cancer changes by adjustment for intake of coffee additives.

## 1. Introduction

Colorectal cancer is one of the most common cancers worldwide, accounting for 10% of all new cancer cases and responsible for 9.4% of all cancer deaths in 2020 [1]. A high incidence and burden of colorectal cancer occurs in eastern Asia [1]. In Korea, colorectal cancer is the most commonly diagnosed cancer and a primary cause of cancer death in men (third most common) and in women (second most common) [2]. Due to the high morbidity and mortality of colorectal cancer, modifiable risk factors for colorectal cancer need to be identified to support guidelines for the prevention of colorectal cancer. Dietary intake is a primary risk factor for colorectal cancer and the consumption of processed or red meat has been suggested to be associated with an increased risk of colorectal cancer in a report from the World Cancer Research Fund [3]. However, colorectal cancer risk associated with the intake of many other foods had only limited evidence, and the results remain inconclusive.

Coffee, which is the most popular beverage worldwide, has been investigated in relation to risks of multiple health outcomes because of its antioxidant and anti-inflammatory properties [4]. Coffee can have a favorable effect on colorectal cancer due to its bioactive components, including polyphenols and melanoidins [5]. Although many observational studies have been performed to examine the association between coffee consumption and the risk of colorectal cancer, the results have been inconsistent, and no study has been performed in a Korean population [6]. Coffee has been the most popular drink and the second most consumed food among Koreans since 2015 [7]. In Korean adults, the intake of instant coffee mix, which includes sugar and cream, account for a high portion of coffee consumption [8]. Coffee additives, including sugar and cream, could contribute to an increased risk of obesity [9,10], which is classified as a strong risk factor for colorectal cancer in the report of the World Cancer Research Fund [3]. To the best of our knowledge, no study has investigated the association between coffee consumption and risk of colorectal cancer considering consumption of coffee additives including sugar and cream. Furthermore, some previous studies showed that the association between the consumption of added sugar and the risk of disease might be different according to the obesity status of subjects [11,12].

In the current study, we analyzed the association between coffee consumption and the risk of colorectal cancer in controls and colorectal cancer patients, and examined whether the association could be changed by adjustment for consumption of coffee additives, including sugar and cream. We hypothesized that the inverse association between coffee consumption and risk of colorectal cancer could be attenuated due to Korean’s high consumption of these coffee additives. Additionally, a stratified analysis by obese status was performed to identify the change in the association between coffee consumption and risk of colorectal cancer by the obesity status of subjects.

## 2. Materials and Methods

### 2.1. Study Population

This hospital-based case-control study was conducted in 2010 to identify the association between dietary intake and risk of colorectal cancer among the Korean population. The cases were defined as patients newly diagnosed with colorectal cancer at the Center for Colorectal Cancer of the National Cancer Center Korea between August 2010 and August 2013 by endoscopic biopsy. The controls were recruited from people visiting the Center for Cancer Prevention and Detection at the National Cancer Center Korea for the health check-up program provided by the National Health Insurance Cooperation between October 2007 and December 2014. Among 15,271 participants (1070 cases and 14,201 controls), the following persons who were not suitable for this study were sequentially excluded: 5189 subjects (145 cases and 5044 controls) with incomplete questionnaires and 122 subjects (2 cases and 120 controls) who had an implausible energy intake (<500 or ≥4000 kcal/day). Of the 9960 eligible subjects (923 cases and 9037 controls), two controls per case were frequency-matched by sex and age (within 5 years) and sex. Finally, 923 cases and 1846 controls were included in this analysis. All participants provided a written informed consent for study participation, and the Institutional Review Board of the National Cancer Center Korea approved the present study guidelines (IRB number: NCCNCS-10-350 and NCC 2015-0202).

### 2.2. Data Collection

Information on demographic and lifestyle factors, including age, sex, socioeconomic status, drinking, smoking, physical activity, and family history of cancer, was collected using a structured questionnaire administered by a trained dietitian. Dietary intake was assessed using a 106-item semi-quantitative food frequency questionnaire (SQFFQ) developed by the Korea Centers for Disease Control and Prevention. The validity and reproducibility of the SQFFQ have been described elsewhere [13]. Each subject was asked to provide an average frequency and a portion size for food items during the preceding year. The food items included coffee and coffee additives, such as sugar and cream. The SQFFQ had 9 frequency levels ranging from ‘none or little’ to ‘three times a day’ and 3 categories for portion size (1/2 serving, 1 serving, 1 and 1/2 servings). Total energy intake was calculated using Computer-Aided Nutritional Analysis Program for Professionals, ver. 4.0 (CAN-Pro 4.0 the Korean Nutrition Society, Seoul, Korea) from the SQFFQ data. Specific clinical information on colorectal cancer was acquired from medical records, and anatomical sub-sites were classified into 3 categories: proximal colon (cecum, ascending colon, hepatic flexure, transverse colon, and splenic flexure), distal colon (descending colon, sigmoid-descending colon junction, sigmoid colon), and rectal (rectosigmoid colon, rectum) [14].

### 2.3. Statistical Analysis

The subjects were divided into 5 categories based on coffee consumption: none, <1 cup/day, 1-<2 cups/day, 2 cups/day, and ≥3 cups/day. The numbers or means of demographic factors and dietary intakes were estimated in each coffee consumption category using linear regression for continuous variables and chi-squared tests for categorical variables. Unconditional logistic regression models were used to calculate the odds ratio (OR) and 95% confidence interval (95% CI) for the association between coffee consumption and the risk of colorectal cancer. We developed the following models: (1) unadjusted; (2) adjusted for age (continuous), sex, education (middle school or lower, high school, and college or higher), occupation (professionals, administrative, management, office jobs/sales or service industry workers/agriculturist, soldier or manufacturing workers/housekeeper, the jobless or others), smoking (non-smoker, ex-smoker, and current smoker), alcohol (non-drinker, ex-drinker, and current drinker), total energy intake (kcal, continuous), physical activity (metabolic equivalent of task [MET] h/week, continuous), body mass index (BMI) (kg/m^2^, continuous), first-degree family history of colorectal cancer (yes/no); and (3) adjusted for covariates in model 2 plus the amounts of coffee additives (sugar and cream) (g/day, continuous). We confirmed whether there is no multicollinearity problem. We assigned a median value for each coffee consumption category to examine linear trends across exposure categories. The association between coffee consumption and risk of colorectal cancer was further explored with stratification by sex, anatomical sub-sites (proximal colon, distal colon, and rectal), and obesity (BMI < 25 and ≥25 kg/m^2^) using a multinomial logistic regression model. All statistical analyses were conducted using SAS software (version 9.4; SAS Institute, Cary, NC, USA) at the 5% significance level.

## 3. Results

### 3.1. General Characteristics of Study Population

The distribution of selected demographic and lifestyle factors for colorectal cancer cases and matched controls is presented in Supplementary Materials Table S1. In both sexes, colorectal cancer cases were more likely to be less educated, have higher total energy intake, or were current smokers, compared with controls.

The general characteristics of the study participants are presented according to coffee consumption in Table 1. People who consumed the most coffee tended to be younger, current smokers, and men; had a lower physical activity, higher BMI, higher education level, and higher energy intake; and consumed more alcohol, compared to those who consumed the least coffee. Participants who drank more coffee consumed more coffee additives including sugar and cream.

Table 2 describes the basic characteristics of the study participants by obese status. Obese participants (BMI ≥ 25 kg/m^2^) tended to be men, were less likely to be non-smokers, and had a higher education level, higher energy intake, and higher alcohol consumption than non-obese participants (BMI < 25 kg/m^2^).

### 3.2. Coffee Consumption and Colorectal Cancer

The ORs and 95% CIs for the association between coffee consumption and the risk of colorectal cancer are presented in Table 3. In model 2, which was adjusted for age, sex, education, occupation, smoking, alcohol, total energy intake, physical activity, BMI, and first-degree family history of colorectal cancer, the OR of colorectal cancer risk for ≥3 cups/day vs. none was 0.68 (95% CI: 0.49–0.96). When we additionally controlled for the consumption of coffee additives (sugar and cream) in model 3, the association was stronger (OR for ≥3 cups/day vs. none = 0.22, 95% CI; 0.14–0.33), and a significant inverse linear trend was observed (*P*_trend_ < 0.0001). In the stratified analysis by anatomical sub-sites, no significant association between coffee consumption and risk of colorectal cancer was observed for the comparison of the highest vs. lowest coffee consumption categories in model 2. However, we found significant inverse associations between coffee consumption and risk of colorectal cancer in model 3 for all anatomical sub-sites. The *p* for trends were 0.0001, 0.0003, and <0.0001 for proximal colon cancer, distal colon cancer, and rectal cancer, respectively. When we investigated the association between coffee consumption and risk of colorectal cancer by sex in model 2, nonsignificant inverse associations were found both in men (*P*_trend_ = 0.74) and women (*P*_trend_ = 0.60). However, the associations became significant in model 3, which was further adjusted for coffee additives (*P*_trend_ < 0.0001 for men and 0.0021 for women). The observed inverse associations between coffee consumption and risk of colorectal cancer were also found for all anatomical sub-sites in model 3 in both men and women, except for distal colon cancer in women. For distal colon cancer in women, a significant inverse association was observed for the comparison of the highest vs. lowest coffee consumption categories (OR for ≥3 cups/day vs. none = 0.29, 95% CI; 0.12–0.74), but the value of *p* for the trend was 0.19, which was not significant.

We performed a stratified analysis by obese status to further evaluate the association between coffee consumption and colorectal cancer risk, and the results are shown in Table 4. We did not observe any significant associations between coffee consumption and colorectal cancer for the comparison of the highest vs. lowest coffee consumption categories in model 2. In model 3, the consumption of three or more cups of coffee was associated with lower odds of developing colorectal cancer, compared to consumption of no coffee in both non-obese people (OR for ≥3 cups/day vs. none = 0.24, 95% CI; 0.14–0.41) and obese people (OR for ≥3 cups/day vs. none = 0.21, 95% CI; 0.10–0.45). In addition, a significant inverse linear trend was observed regardless of obesity status (*P*_trend_ < 0.0001 for non-obese people and obese people). The inverse associations between coffee consumption and colorectal cancer among non-obese people and obese people were consistent irrespective of anatomical sub-sites. The *p* for trend was < 0.02 for all anatomical sub-sites among both non-obese people and obese people.

## 4. Discussion

In the present study, we investigated whether the association between coffee consumption and the risk of colorectal cancer was altered by adjustment for coffee additives, such as sugar and cream. We found that people who consumed ≥3 cups/day of coffee had 32% lower odds of developing colorectal cancer than non-coffee drinkers. The observed inverse association between coffee consumption and the risk of colorectal cancer became stronger when we controlled for consumption of coffee additives including sugar and cream, indicating 78% lower odds of developing colorectal cancer, and a significant inverse linear trend was also shown between coffee consumption and risk of colorectal cancer. In the analyses stratified by sex, anatomical sub-sites, and obesity status, the nonsignificant inverse association between coffee consumption and risk of colorectal cancer became significant after adjustment for intake of coffee additives.

Two previous meta-analyses investigating the association of coffee consumption with colorectal cancer risk reported that high coffee consumption was associated with 30% [15] and 15% [6] decreased odds of developing colorectal cancer compared to low coffee consumption among case-control studies, respectively. The results from prospective cohort studies mostly showed suggestive inverse associations indicating borderline significance. The relative risks for the highest vs. lowest coffee consumption were 0.91 (95% CI: 0.81–1.02) in a meta-analysis of 12 prospective cohort studies [16] and 0.94 (95% CI: 0.88–1.01) in a meta-analysis of 16 prospective cohort studies [6]. Two recent large case-control studies also reported reduced risks of colorectal cancer in people with high coffee consumption [17,18]. Our results from the Korean population are consistent with previous studies in that they showed an inverse association between coffee consumption and the risk of colorectal cancer. However, we observed a greater reduction in the odds of developing colorectal cancer than previous studies. The difference in the degree of decrease in the odds of developing colorectal cancer seems to be due to adjustment for consumption of coffee additives, such as sugar and cream. Among the previous studies reporting the association between coffee consumption and colorectal cancer risk, no study controlled for the intake of coffee additives.

Changes in odds values after adjustment for intake of coffee additives were also observed in the analysis stratified by sex and anatomical sub-sites. In model 2, we found a slightly stronger inverse association between coffee consumption and colorectal cancer risk in women than in men. This result was similar to the results of previous studies indicating greater decrease in the odds or risks of colorectal cancer in women than in men [6,15]. However, men showed lower odds of developing colorectal cancer than women in model 3, which was further adjusted for intake of coffee additives. Regarding anatomical sub-sites, rectal cancer showed a weaker inverse association in relation to coffee consumption than proximal or distal colon cancer in model 2; this is consistent with previous evidence [6,15]. After adjustment for intake of coffee additives, the inverse association became similar irrespective of anatomical sub-sites, showing greater reductions in the odds of developing cancer. When we analyzed by the obesity status of subjects, a decrease in the odds of developing colorectal cancer was also observed as a result of additional adjustment for intake of coffee additives. Furthermore, the degree of reduction in the odds was different based on the obesity status of the subjects. A greater decrease in the odds of developing cancer was observed in obese people than in non-obese people. Thus, we found a slightly stronger inverse association between coffee consumption and the risk of colorectal cancer in obese people than in non-obese people. Previous studies reporting the association between coffee consumption and risk of disease by the BMI of subjects showed inconsistent results. Regarding the risk of mortality, overweight subjects with high coffee consumption had a decreased risk of death than normal-weight subjects [19]. However, a higher risk of type 2 diabetes was observed among subjects with high BMI than among those with low BMI [20,21]. Given the evidence to date, further studies are warranted to explore differences in the association between coffee consumption and the risk of disease according to the obesity status of subjects.

Several potential mechanisms can explain the inverse association between coffee consumption and the risk of colorectal cancer. First, coffee components could reduce the synthesis [22] and secretion [23] of bile acids, which have been found to be promotors of colon cancer [24,25] through downregulation of the expression of bile acid homeostatic genes [23]. Second, coffee has also been reported to increase colonic motility, especially in the rectosigmoid region, and thus may decrease the risk of colorectal cancer [5,26] by reducing the exposure of epithelial cells to carcinogens [5]. Third, coffee includes several antioxidant and antimutagenic constituents, including phenolic compounds (chlorogenic, caffeic, ferulic, and cumaric acids), diterpenes (cafestol and kahweol), and melanoidins [27,28]. Among these ingredients of coffee, cafestol and kahweol have been reported to decrease the genotoxicity of some carcinogens [28,29]. Additionally, chlorogenic acids have been observed to improve glucose metabolism through the activation of AMP-dependent kinase (AMPK) and hindering hepatic glucose-6-phosphatase expression and activity [30,31]. Eventually, these functions of chlorogenic acids could contribute to reducing the risk of colorectal cancer because type 2 diabetes is known as a risk factor for colorectal cancer [32,33].

To the best of our knowledge, this is the first case-control study to investigate the association between coffee consumption and the risk of colorectal cancer in a Korean population. We additionally conducted an analysis adjusting for intakes of coffee sugar and coffee cream, considering that many Koreans usually drink coffee with sugar and cream. Our results indicating a greater decrease in the odds of developing colorectal cancer than previous studies suggest that future studies investigating the association between coffee consumption and risk of colorectal cancer need to consider additionally adjusting for consumption of coffee additives, such as sugar and cream. There are several limitations to be acknowledged in this study. This study might have some degree of selection bias due to the hospital-based case-control study design. People in the control group may be more sensitive to health and more likely to practice a healthy lifestyle than those in the case group. In case-control studies, recall bias, which indicates that subjects in the case group may tend to remember food intakes better than those in the control group, can occur. In addition, observational studies cannot be free from some residual and unmeasured confounding factors, although we adjusted for many potential risk factors of colorectal cancer, including BMI, smoking, alcohol consumption, and physical activity. Finally, our results cannot be generalized because this study targeted only the Korean population.

## 5. Conclusions

In conclusion, we found that high coffee consumption was associated with decreased odds of developing colorectal cancer. The observed inverse association between coffee consumption and colorectal cancer became stronger after additional adjustment for consumption of coffee additives, including sugar and cream. The inverse linear trends for coffee consumption and colorectal cancer were also maintained in the analyses stratified by the sex, anatomical sub-sites, and obesity status of the subjects. Future large prospective cohort studies that control for intake of coffee additives should be conducted in various geographic regions to identify the benefits of coffee drinking in relation to colorectal cancer risk.

## Figures and Tables

**Table 1:** Characteristics of the study subjects according to coffee consumption, N (%).

	Coffee Consumption					
	None	<1 Cup/Day	1-<2 Cups/Day	2 Cups/Day	≥3 Cups/Day	*p*-Value ^b^
***n***	314	635	620	554	646	
**Age ^a^, years**	57.81 ± 9.66	56.60 ± 9.76	57.68 ± 9.09	55.30 ± 9.15	54.61 ± 8.72	<0.0001
**Sex**						
Male	177 (56.4)	422 (66.5)	389 (62.7)	380 (68.6)	507 (78.5)	<0.0001
Female	137 (43.6)	213 (33.5)	231 (37.3)	174 (31.4)	139 (21.5)	
**BMI ^a^, kg/m^2^**	23.60 ± 3.29	24.26 ± 3.00	24.13 ± 3.08	24.27 ± 2.83	24.33 ± 3.17	0.0087
**Physical activity, MET h/week**						
<5	40 (12.7)	68 (10.7)	65 (10.5)	86 (15.5)	95 (14.7)	0.22
5-<20	75 (23.9)	171 (26.9)	152 (24.5)	123 (22.2)	153 (23.7)	
20-<50	97 (30.9)	200 (31.5)	213 (34.4)	167 (30.1)	206 (31.9)	
≥50	102 (32.5)	196 (30.9)	190 (30.7)	178 (32.1)	192 (29.7)	
**Education level**						
Middle school or lower	106 (34.3)	130 (20.9)	141 (23.1)	106 (19.4)	120 (18.8)	<0.0001
High school	114 (36.9)	207 (33.3)	205 (33.6)	188 (34.4)	242 (37.9)	
College or higher	89 (28.8)	285 (45.8)	264 (43.3)	253 (46.3)	276 (43.3)	
**Smoking status**						
Non-smoker	194 (61.8)	322 (50.7)	310 (50.0)	222 (40.1)	179 (27.7)	<0.0001
Ex-smoker	88 (28.0)	216 (34.0)	217 (35.0)	228 (41.2)	256 (39.6)	
Current smoker	32 (10.2)	97 (15.3)	93 (15.0)	104 (18.8)	211 (32.7)	
**Alcohol consumption**						
Non-drinker	144 (45.9)	204 (32.1)	195 (31.5)	140 (25.3)	156 (24.2)	<0.0001
Ex-drinker	30 (9.6)	68 (10.7)	53 (8.6)	60 (10.8)	87 (13.5)	
Current drinker	140 (44.6)	363 (57.2)	372 (60.0)	354 (63.9)	403 (62.4)	
**Occupation**						
Professionals, administrative, management, or office jobs	58 (18.7)	158 (25.2)	124 (20.2)	149 (27.1)	181 (28.2)	<0.0001
Sales or service industry workers	30 (9.7)	90 (14.4)	96 (15.6)	103 (18.7)	122 (19.0)	
Agriculturist, soldier, or manufacturing workers	38 (12.2)	71 (11.3)	83 (13.5)	72 (13.1)	118 (18.4)	
Housekeeper, the jobless or others	185 (59.5)	308 (49.1)	312 (50.7)	226 (41.1)	222 (34.5)	
**First-degree family history of CRC**						
Yes	20 (6.4)	43 (6.8)	42 (6.8)	34 (6.1)	46 (7.1)	0.97
No	293 (93.6)	591 (93.2)	576 (93.2)	520 (93.9)	600 (92.9)	
**Energy intake ^a^, kcal/day**	1730.36 ± 574.47	1674.45 ± 533.35	1778.75 ± 570.83	1812.26 ± 519.85	1975.05 ± 617.16	<0.0001
**Coffee additives**						
Coffee sugar ^a^, g/day	0.00 ± 0.00	1.20 ± 1.68	3.21 ± 2.45	5.69 ± 4.29	9.29 ± 7.20	<0.0001
Coffee cream ^a^, g/day	0.00 ± 0.00	0.69 ± 1.05	2.08 ± 2.01	3.92 ± 3.42	6.36 ± 5.86	<0.0001

MET, metabolic equivalent of task. ^a^ Values are means ± SD. ^b^ *p*-values were derived from the chi square test for categorical variables and linear regression for a continuous variable.

**Table 2:** Characteristics of the study subjects according to obese status, N (%).

	Obesity Status		
	BMI < 25 kg/m^2^	BMI ≥ 25 kg/m^2^	*p*-Value ^b^
***n***	1766	1003	
**Age ^a^, years**	56.08 ± 9.35	56.57 ± 9.26	0.18
**Sex**			
Male	1140 (64.6)	735 (73.3)	<0.0001
Female	626 (35.5)	268 (26.7)	
**BMI ^a^, kg/m^2^**	22.41 ± 1.87	27.29 ± 2.13	<0.0001
**Physical activity, MET h/week**			
<5	219 (12.4)	135 (13.5)	0.49
5-<20	443 (25.1)	231 (23.0)	
20-<50	552 (31.3)	331 (33.0)	
≥50	552 (31.3)	306 (30.5)	
**Education level**			
Middle school or lower	370 (21.3)	233 (23.6)	0.0047
High school	649 (37.3)	307 (31.1)	
College or higher	720 (41.4)	447 (45.3)	
**Smoking status**			
Non-smoker	835 (47.3)	392 (39.1)	<0.0001
Ex-smoker	586 (33.2)	419 (41.8)	
Current smoker	345 (19.5)	192 (19.1)	
**Alcohol consumption**			
Non-drinker	568 (32.2)	271 (27.0)	0.02
Ex-drinker	183 (10.4)	115 (11.5)	
Current drinker	1015 (57.5)	617 (61.5)	
**Occupation**			
Professionals, Administrative, Management, Office jobs	406 (23.2)	264 (26.5)	0.0038
Sales or service industry workers	261 (14.9)	180 (18.1)	
Agriculturist, soldier, or manufacturing workers	240 (13.7)	142 (14.2)	
Housekeeper, the jobless or others	842 (48.1)	411 (41.2)	
**First-degree family history of CRC**			
Yes	116 (6.6)	69 (6.9)	0.75
No	1648 (93.4)	932 (93.1)	
**Energy intake ^a^, kcal/day**	1770.21 ± 556.64	1857.55 ± 599.72	0.0001
**Coffee additives**			
Coffee sugar ^a^, g/day	4.22 ± 5.25	4.43 ± 5.50	0.32
Coffee cream ^a^, g/day	2.80 ± 3.98	3.07 ± 4.28	0.10

MET, metabolic equivalent of task. ^a^ Values are means ± SD. ^b^ *p*-values were derived from the chi square test for categorical variables and linear regression for a continuous variable.

**Table 3:** Association between coffee consumption and colorectal cancer (odds ratios and 95% confidence intervals).

	Coffee Consumption					
	None	<1 Cup/Day	1-<2 Cups/Day	2 Cups/Day	≥3 Cups/Day	*p*-Trend
**Overall**						
No. of controls	173	470	405	389	409	
**Colorectal cancer**						
No. of cases	141	165	215	165	237	
Model 1 ^a^	1.0 (ref)	0.43 (0.32–0.57)	0.65 (0.49–0.86)	0.52 (0.39–0.69)	0.71 (0.54–0.94)	0.68
Model 2 ^b^	1.0 (ref)	0.52 (0.37–0.72)	0.79 (0.57–1.09)	0.60 (0.42–0.84)	0.68 (0.49–0.96)	0.48
Model 3 ^c^	1.0 (ref)	0.44 (0.32–0.62)	0.53 (0.38–0.74)	0.29 (0.20–0.42)	0.22 (0.14–0.33)	<0.0001
**Proximal colon cancer**						
No. of cases	32	38	33	23	39	
Model 1 ^a^	1.0 (ref)	0.44 (0.27–0.72)	0.44 (0.26–0.74)	0.32 (0.18–0.56)	0.52 (0.31–0.85)	0.13
Model 2 ^b^	1.0 (ref)	0.55 (0.31–0.96)	0.58 (0.32–1.03)	0.39 (0.21–0.73)	0.56 (0.31–1.01)	0.12
Model 3 ^c^	1.0 (ref)	0.49 (0.28–0.85)	0.43 (0.24–0.78)	0.23 (0.11–0.45)	0.21 (0.10–0.47)	0.0001
**Distal colon cancer**						
No. of cases	45	49	65	64	71	
Model 1 ^a^	1.0 (ref)	0.40 (0.26–0.62)	0.62 (0.41–0.94)	0.63 (0.42–0.96)	0.67 (0.44–1.01)	0.63
Model 2 ^b^	1.0 (ref)	0.48 (0.29–0.78)	0.77 (0.48–1.23)	0.69 (0.42–1.11)	0.69 (0.43–1.13)	0.98
Model 3 ^c^	1.0 (ref)	0.42 (0.26–0.69)	0.56 (0.34–0.90)	0.36 (0.21–0.62)	0.25 (0.14–0.47)	0.0003
**Rectal cancer**						
No. of cases	62	73	113	75	121	
Model 1 ^a^	1.0 (ref)	0.43 (0.30–0.63)	0.78 (0.55–1.11)	0.54 (0.37–0.79)	0.83 (0.58–1.18)	0.28
Model 2 ^b^	1.0 (ref)	0.54 (0.35–0.83)	0.90 (0.59–1.36)	0.61 (0.40–0.95)	0.77 (0.50–1.19)	0.86
Model 3 ^c^	1.0 (ref)	0.45 (0.29–0.69)	0.59 (0.38–0.90)	0.27 (0.17–0.45)	0.21 (0.12–0.37)	<0.0001
**Male**						
No. of controls	98	313	250	271	318	
**Colorectal cancer**						
No. of cases	79	109	139	109	189	
Model 1 ^a^	1.0 (ref)	0.43 (0.30–0.62)	0.69 (0.48–0.99)	0.50 (0.34–0.72)	0.74 (0.52–1.04)	0.41
Model 2 ^b^	1.0 (ref)	0.63 (0.40–0.99)	0.96 (0.61–1.53)	0.71 (0.44–1.13)	0.78 (0.49–1.23)	0.74
Model 3 ^c^	1.0 (ref)	0.53 (0.33–0.83)	0.63 (0.40–1.01)	0.31 (0.19–0.52)	0.20 (0.11–0.36)	<0.0001
**Proximal colon cancer**						
No. of cases	19	25	18	17	33	
Model 1 ^a^	1.0 (ref)	0.41 (0.22–0.78)	0.37 (0.19–0.74)	0.32 (0.16–0.65)	0.54 (0.29–0.98)	0.55
Model 2 ^b^	1.0 (ref)	0.70 (0.33–1.48)	0.62 (0.28–1.37)	0.57 (0.25–1.27)	0.72 (0.34–1.53)	0.59
Model 3 ^c^	1.0 (ref)	0.63 (0.30–1.33)	0.49 (0.22–1.11)	0.36 (0.15–0.87)	0.31 (0.11–0.82)	0.02
**Distal colon cancer**						
No. of cases	20	32	42	35	51	
Model 1 ^a^	1.0 (ref)	0.50 (0.27–0.92)	0.82 (0.46–1.47)	0.63 (0.35–1.15)	0.79 (0.45–1.38)	0.64
Model 2 ^b^	1.0 (ref)	0.71 (0.36–1.42)	1.11 (0.57–2.19)	0.83 (0.41–1.66)	0.83 (0.42–1.65)	0.80
Model 3 ^c^	1.0 (ref)	0.61 (0.31–1.21)	0.77 (0.39–1.54)	0.38 (0.18–0.82)	0.22 (0.09–0.54)	0.0005
**Rectal cancer**						
No. of cases	38	48	78	55	101	
Model 1 ^a^	1.0 (ref)	0.40 (0.24–0.64)	0.81 (0.51–1.27)	0.52 (0.33–0.84)	0.82 (0.53–1.27)	0.19
Model 2 ^b^	1.0 (ref)	0.61 (0.34–1.09)	1.06 (0.60–1.87)	0.79 (0.44–1.41)	0.91 (0.52–1.62)	0.65
Model 3 ^c^	1.0 (ref)	0.49 (0.28–0.88)	0.64 (0.36–1.14)	0.29 (0.15–0.57)	0.17 (0.08–0.37)	<0.0001
**Female**						
No. of controls	75	157	155	118	91	
**Colorectal cancer**						
No. of cases	62	56	76	56	48	
Model 1 ^a^	1.0 (ref)	0.43 (0.27–0.68)	0.59 (0.38–0.92)	0.57 (0.36–0.91)	0.64 (0.39–1.04)	0.83
Model 2 ^b^	1.0 (ref)	0.45 (0.27–0.76)	0.69 (0.42–1.13)	0.58 (0.34–0.99)	0.60 (0.34–1.07)	0.60
Model 3 ^c^	1.0 (ref)	0.38 (0.23–0.64)	0.44 (0.26–0.75)	0.28 (0.15–0.52)	0.25 (0.12–0.49)	0.0021
**Proximal colon cancer**						
No. of cases	13	13	15	6	6	
Model 1 ^a^	1.0 (ref)	0.48 (0.21–1.08)	0.56 (0.25–1.23)	0.29 (0.11–0.81)	0.38 (0.14–1.05)	0.07
Model 2 ^b^	1.0 (ref)	0.42 (0.17–1.03)	0.58 (0.24–1.41)	0.25 (0.08–0.78)	0.27 (0.08–0.91)	0.05
Model 3 ^c^	1.0 (ref)	0.37 (0.15–0.93)	0.37 (0.14–0.98)	0.13 (0.04–0.44)	0.12 (0.03–0.54)	0.0047
**Distal colon cancer**						
No. of cases	25	17	23	29	20	
Model 1 ^a^	1.0 (ref)	0.33 (0.17–0.64)	0.45 (0.24–0.84)	0.74 (0.40–1.35)	0.66 (0.34–1.28)	0.38
Model 2 ^b^	1.0 (ref)	0.34 (0.16–0.72)	0.58 (0.29–1.16)	0.70 (0.35–1.42)	0.62 (0.29–1.34)	0.72
Model 3 ^c^	1.0 (ref)	0.29 (0.14–0.62)	0.40 (0.19–0.83)	0.39 (0.18–0.87)	0.29 (0.12–0.74)	0.19
**Rectal cancer**						
No. of cases	24	25	35	20	20	
Model 1 ^a^	1.0 (ref)	0.50 (0.27–0.93)	0.71 (0.39–1.27)	0.53 (0.27–1.03)	0.69 (0.35–1.34)	0.68
Model 2 ^b^	1.0 (ref)	0.45 (0.23–0.91)	0.75 (0.39–1.46)	0.49 (0.24–1.04)	0.60 (0.28–1.29)	0.55
Model 3 ^c^	1.0 (ref)	0.36 (0.18–0.74)	0.50 (0.25–1.01)	0.27 (0.12–0.62)	0.25 (0.10–0.64)	0.02

^a^ Model 1 was unadjusted. ^b^ Model 2 adjusted for age, sex (only in overall), education, occupation, smoking, alcohol, total energy intake, physical activity, BMI, first-degree family history of CRC. ^c^ Model 3 was adjusted for covariates in Model 2 plus coffee additives (sugar, cream).

**Table 4:** Association between coffee consumption and colorectal cancer by obese status (odds ratios and 95% confidence intervals).

	Coffee Consumption					
	None	<1 Cup/Day	1-<2 Cups/Day	2 Cups/Day	≥3 Cups/Day	*p*-Trend
**BMI < 25 kg/m^2^**						
**Colorectal cancer**						
No. of cases	106	101	143	100	148	
No. of controls	118	302	260	243	245	
Model 1 ^a^	1.0 (ref)	0.37 (0.26–0.53)	0.61 (0.44–0.85)	0.46 (0.32–0.65)	0.67 (0.48–0.94)	0.95
Model 2 ^b^	1.0 (ref)	0.44 (0.29–0.67)	0.75 (0.49–1.13)	0.58 (0.38–0.90)	0.70 (0.45–1.07)	0.97
Model 3 ^c^	1.0 (ref)	0.38 (0.25–0.58)	0.51 (0.33–0.78)	0.28 (0.17–0.46)	0.24 (0.14–0.41)	<0.0001
**Proximal colon cancer**						
No. of cases	25	21	22	14	23	
No. of controls	118	302	260	243	245	
Model 1 ^a^	1.0 (ref)	0.33 (0.18–0.61)	0.40 (0.22–0.74)	0.27 (0.14–0.54)	0.44 (0.24–0.81)	0.12
Model 2 ^b^	1.0 (ref)	0.44 (0.22–0.89)	0.57 (0.28–1.16)	0.44 (0.20–0.95)	0.60 (0.29–1.25)	0.485
Model 3 ^c^	1.0 (ref)	0.38 (0.19–0.78)	0.4 (0.19–0.84)	0.24 (0.10–0.56)	0.22 (0.08–0.58)	0.0066
**Distal colon cancer**						
No. of cases	30	30	39	34	39	
No. of controls	118	302	260	243	245	
Model 1 ^a^	1.0 (ref)	0.39 (0.23–0.68)	0.59 (0.35–1.00)	0.55 (0.32–0.94)	0.63 (0.37–1.06)	0.93
Model 2 ^b^	1.0 (ref)	0.50 (0.26–0.94)	0.80 (0.43–1.49)	0.77 (0.41–1.45)	0.72 (0.38–1.38)	0.91
Model 3 ^c^	1.0 (ref)	0.44 (0.23–0.83)	0.59 (0.31–1.11)	0.41 (0.20–0.83)	0.28 (0.13–0.64)	0.01
**Rectal cancer**						
No. of cases	49	47	79	50	81	
No. of controls	118	302	260	243	245	
Model 1 ^a^	1.0 (ref)	0.38 (0.24–0.59)	0.73 (0.48–1.11)	0.50 (0.32–0.78)	0.80 (0.53–1.21)	0.38
Model 2 ^b^	1.0 (ref)	0.45 (0.26–0.77)	0.87 (0.53–1.46)	0.62 (0.36–1.08)	0.84 (0.50–1.44)	0.51
Model 3 ^c^	1.0 (ref)	0.38 (0.22–0.65)	0.59 (0.35–0.99)	0.29 (0.16–0.54)	0.27 (0.14–0.53)	0.0013
**BMI** **≥ 25 kg/m^2^**						
**Colorectal cancer**						
No. of cases	35	64	72	65	89	
No. of controls	55	168	145	146	164	
Model 1 ^a^	1.0 (ref)	0.60 (0.36–1.00)	0.78 (0.47–1.30)	0.70 (0.42–1.17)	0.85 (0.52–1.40)	0.50
Model 2 ^b^	1.0 (ref)	0.82 (0.46–1.47)	1.00 (0.56–1.78)	0.78 (0.43–1.41)	0.83 (0.46–1.51)	0.58
Model 3 ^c^	1.0 (ref)	0.69 (0.39–1.24)	0.66 (0.36–1.21)	0.35 (0.18–0.67)	0.21 (0.10–0.45)	<0.0001
**Proximal colon cancer**						
No. of cases	7	17	11	9	16	
No. of controls	55	168	145	146	164	
Model 1 ^a^	1.0 (ref)	0.80 (0.31–2.02)	0.60 (0.22–1.62)	0.48 (0.17–1.36)	0.77 (0.30–1.96)	0.62
Model 2 ^b^	1.0 (ref)	0.85 (0.31–2.34)	0.66 (0.22–1.95)	0.39 (0.13–1.20)	0.59 (0.20–1.75)	0.17
Model 3 ^c^	1.0 (ref)	0.77 (0.28–2.14)	0.54 (0.18–1.65)	0.25 (0.07–0.83)	0.25 (0.06–0.96)	0.01
**Distal colon cancer**						
No. of cases	15	19	26	30	32	
No. of controls	55	168	145	146	164	
Model 1 ^a^	1.0 (ref)	0.42 (0.20–0.87)	0.66 (0.32–1.33)	0.75 (0.38–1.51)	0.72 (0.36–1.42)	0.46
Model 2 ^b^	1.0 (ref)	0.54 (0.24–1.22)	0.82 (0.37–1.80)	0.76 (0.34–1.67)	0.73 (0.33–1.63)	0.96
Model 3 ^c^	1.0 (ref)	0.48 (0.21–1.08)	0.59 (0.26–1.34)	0.38 (0.16–0.90)	0.21 (0.07–0.59)	0.0071
**Rectal cancer**						
No. of cases	13	26	34	25	40	
No. of controls	55	168	145	146	164	
Model 1 ^a^	1.0 (ref)	0.66 (0.32–1.36)	0.99 (0.49–2.02)	0.72 (0.35–1.52)	1.03 (0.51–2.07)	0.41
Model 2 ^b^	1.0 (ref)	0.87 (0.39–1.96)	1.09 (0.49–2.41)	0.76 (0.33–1.73)	0.90 (0.40–2.04)	0.70
Model 3 ^c^	1.0 (ref)	0.69 (0.31–1.55)	0.62 (0.27–1.42)	0.27 (0.11–0.68)	0.14 (0.05–0.43)	<0.0001

^a^ Model 1 was unadjusted. ^b^ Model 2 adjusted for age, sex, education, occupation, smoking, alcohol, total energy intake, physical activity, BMI, first-degree family history of CRC. ^c^ Model 3 was adjusted for covariates in Model 2 plus coffee additives (sugar, cream).

## Data Availability

Not applicable.

## Supplementary Materials

The following are available online at https://www.mdpi.com/article/10.3390/nu13082753/s1, Table S1: Characteristics of cases and controls.
