# Supplementary material for: The Association between Coffee Consumption and Risk of Colorectal Cancer in a Korean Population
Source: Nutrients. 2021 Aug 11;13(8):2753. doi: 10.3390/nu13082753 (PMC8400085; doi:10.3390/nu13082753)
Supplement: Supplementary file 1 [file nutrients-13-02753-s001.zip › modified_nutrients-1304506 - supple.pdf]

Supplementary Table S1. Characteristics of cases and control

	Total			Male			Female		
	**case**	**control**	***P*-value[b)]**	**case**	**control**	***P*-value[b)]**	**case**	**control**	***P*-value[b)]**
**n**	923	1846		625	1250		298	596	
**Age[a)], years**	56.58±9.71	56.09±9.12	0.1996	57.2±9.4	56.63±8.73	0.2046	55.28±10.23	54.95±9.79	0.6426
**BMI[a)], kg/m^2**	24.00±3.44	24.26±2.86	0.0502	23.93±0.13	24.61±0.08	<.0001	24.16±3.99	23.53±2.97	0.0164
**Physical activity, MET h/week**									
<5	78 (8.5)	276 (15.0)	<.0001	50 (8.0)	168 (13.4)	<.0001	28 (9.40)	108 (18.12)	0.002
5-<20	251 (27.2)	423 (22.9)		163 (26.1)	240 (19.2)		88 (29.53)	183 (30.70)	
20-<50	341 (36.9)	542 (29.4)		227 (36.3)	366 (29.3)		114 (38.26)	176 (29.53)	
≥50	253 (27.4)	605 (32.8)		185 (29.6)	476 (38.1)		68 (22.82)	129 (21.64)	
**Education level**									
Middle school or lower	321 (34.8)	282 (15.6)	<.0001	183 (29.3)	175 (14.4)	<.0001	138 (46.31)	107 (18.23)	<.0001
High school	369 (40.0)	587 (32.6)		266 (42.6)	329 (27.1)		103 (34.56)	258 (43.95)	
College or higher	233 (25.2)	934 (51.8)		176 (28.2)	712 (58.6)		57 (19.13)	222 (37.82)	
**Smoking status**									
Non-smoker	409 (44.31)	818 (44.31)	0.156	145 (23.2)	247 (19.8)	0.082	264 (88.59)	571 (95.81)	<.0001
Ex-smoker	318 (34.45)	687 (37.22)		303 (48.5)	671 (53.7)		15 (5.03)	16 (2.68)	
Current smoker	196 (21.24)	341 (18.47)		177 (28.3)	332 (26.6)		19 (6.38)	9 (1.51)	
**Alcohol consumption**									
Non-drinker	279 (30.23)	560 (30.34)	0.0004	107 (17.12)	200 (16.00)	0.0013	172 (57.72)	360 (60.40)	0.1906
Ex-drinker	129 (13.98)	169 (9.15)		103 (16.48)	136 (10.88)		26 (8.72)	33 (5.54)	
Current drinker	515 (55.8)	1117 (60.51)		415 (66.40)	914 (73.12)		100 (33.56)	203 (34.06)	
**Occupation**									
Professionals, administrative, management, or office jobs	189 (20.48)	481 (26.39)	<.0001	160 (25.60)	389 (31.57)	<.0001	29 (9.73)	92 (15.57	<.0001
Sales or service industry workers	38 (4.12)	403 (22.11)		28 (4.48)	308 (25.00)		10 (3.36)	95 (16.07)	
Agriculturist, soldier or manufacturing workers	141 (15.28)	241 (13.22)		121 (19.36)	221 (17.94)		20 (6.71)	20 (3.38)	
Housekeeper, the jobless or others	555 (60.13)	698 (38.29)		316 (50.56)	314 (25.49)		239 (80.20)	384 (64.97)	

**First-degree family history of colorectal cancer**									
Yes	86 (9.32)	99 (5.37)	<.0001	65 (10.40)	58 (4.65)	<.0001	21 (7.05)	41 (6.88)	0.9258
No	837 (90.68)	1743 (94.63)		560 (89.60)	1188 (95.35)		277 (92.95)	555 (93.12)	
**Energy intake[a)], kcal/d**	2026.3±534	1689.6±560.4	<.0001	2127.4±509.1	1730.4±547.2	<.0001	1814.4±523.5	1604.0±578.4	<.0001
**Coffee additives**									
Coffee sugar[a)], g/day	5.93±6.35	3.49±4.55	<.0001	6.78±6.82	3.93±4.82	<.0001	4.14±4.76	2.55±3.74	<.0001
Coffee cream[a)], g/day	4.22±4.94	2.23±3.40	<.0001	4.93±5.34	2.52±3.57	<.0001	2.73±3.54	1.62±2.95	<.0001

MET, metabolic equivalent of task

[a] Values are means ± SD

[b] *P*-values were derived from the chi square test for categorical variables and linear regression for a continuous variable
